# Neonatal Mortality Rates With and Without a Minimum Threshold

**DOI:** 10.1001/jamanetworkopen.2024.47487

**Published:** 2024-11-22

**Authors:** Fu-Wen Liang, Yu-Shan Chang, Chia-Sui Chou, Mei-Jy Jeng, Ichiro Kawachi, Tsung-Hsueh Lu

**Affiliations:** 1 Department of Public Health, College of Health Sciences, Kaohsiung Medical University, Kaohsiung, Taiwan; 2 Department of Medical Research, Kaohsiung Medical University Hospital, Kaohsiung, Taiwan; 3 Center for Big Data Research, Kaohsiung Medical University, Kaohsiung, Taiwan; 4 Department of Emergency Medicine, Chi Mei Medical Center, Tainan, Taiwan; 5 Neonatal Medical Care Center, Department of Pediatrics, Taipei Veterans General Hospital, Taipei, Taiwan; 6 Institute of Emergency and Critical Care Medicine, College of Medicine, National Yang Ming Chiao Tung University, Taipei, Taiwan; 7 Department of Social and Behavioral Sciences, Harvard T.H. Chan School of Public Health, Boston, Massachusetts; 8 Department of Public Health, College of Medicine, National Cheng Kung University, Tainan, Taiwan

## Abstract

This cross-sectional study examines international variations in neonatal mortality rates with and without minimum thresholds for gestational age (22 weeks) and birth weight (500 g) in 2010 and 2020.

## Introduction

International comparison of neonatal mortality rates (NMRs) is important but challenging owing to variations in criteria for registering live births and obstetricians’ practices for registering live births for periviable newborns across countries.^1,2,3,4^ To address this problem, Organisation for European Economic Co-operation and Development (OECD) Health Statistics provides NMRs with and without a minimum threshold (MT) of 22 weeks’ gestational age or a birth weight of 500 g. However, little is known regarding the magnitude of differences between NMRs according to the 2 definitions across countries and years. This study examined international variations in NMRs with and without an MT in 2010 and 2020.

## Methods

In this cross-sectional study, we obtained NMRs (deaths per 1000 live births) based on the 2 definitions from OECD Health Statistics for 2010 and 2020 (eAppendix in Supplement 1). Data regarding the number of live births and neonatal deaths without an MT for each country were extracted from the World Health Organization Mortality Database (eAppendix in Supplement 1); Taiwan data were taken from Lu et al.^5^ We calculated 95% CIs for NMRs, differences, and ratios between NMRs with and without an MT for each country (formulas for calculating 95% CIs are shown in the eAppendix in Supplement 1). At a *P* < .05 significance level, differences, and ratios between NMRs with and without an MT were considered significant if the 95% CI did not include 0.0 and 1.0, respectively. Data analysis was performed from December 18 to December 25, 2023. Since the study used publicly available, deidentified data, patient informed consent was waived in accordance with 45 CFR §46. This study protocol was approved by National Cheng Kung University Hospital. We followed the STROBE reporting guidelines.

## Results

Our analysis included 12 988 445 live births and 37 620 neonatal deaths in 30 countries in 2010, and 8 039 571 live births and 21 820 neonatal deaths in 26 countries in 2020. Six countries (Austria, New Zealand, Germany, Taiwan, United Kingdom, and the US) in 2010 and 4 countries (Austria, Taiwan, Switzerland, and the US) in 2020 exhibited significant differences in NMRs with and without an MT (Table 1 and Table 2). By contrast, there were 12 countries in 2010 and 8 countries in 2020 that revealed the same NMRs between those with and without an MT.

**Table 1.** NMRs, Country Ranks, Differences, and Ratios Between NMRs Without and With MTs for 30 Countries in 2010^a^

Country	Births, No.	Without MT		With MT		Difference between NMRs (95% CI)	Ratio of NMRs (95% CI)
		NMR (95% CI)	Rank	NMR (95% CI)	Rank		
Australia	297 903	2.8 (2.6 to 3.0)	19	2.8 (2.6 to 3.0)	22	0.0 (−0.3 to 0.2)	1.0 (0.9 to 1.1)
Austria	78 742	2.7 (2.3 to 3.1)	18	2.2 (1.9 to 2.5)	14	−0.5 (−1.0 to −0.0)^b^	0.8 (0.7 to 1.0)^b^
Belgium	130 100	2.2 (1.9 to 2.5)	12	2.2 (1.9 to 2.5)	14	0.0 (−0.4 to 0.3)	1.0 (0.9 to 1.2)
Chile	251 569	5.1 (4.8 to 5.4)	29	4.8 (4.5 to 5.1)	29	−0.3 (−0.7 to 0.1)	0.9 (0.9 to 1.0)
Colombia	654 935	7.7 (7.5 to 7.9)	30	7.4 (7.2 to 7.6)	30	−0.3 (−0.6 to 0.0)	1.0 (0.9 to 1.0)
Czechia	117 153	1.7 (1.5 to 1.9)	5	1.7 (1.5 to 1.9)	7	0.0 (−0.3 to 0.4)	1.0 (0.8 to 1.2)
Denmark	63 411	2.4 (2.0 to 2.8)	14	1.9 (1.6 to 2.2)	11	−0.5 (−1.0 to 0.0)	0.8 (0.6 to 1.0)
Estonia	15 825	1.9 (1.2 to 2.6)	10	1.8 (1.1 to 2.5)	8	−0.1 (−1.0 to 0.9)	1.0 (0.6 to 1.6)
Finland	60 980	1.5 (1.2 to 1.8)	3	1.5 (1.2 to 1.8)	4	0.0 (−0.4 to 0.4)	1.0 (0.8 to 1.3)
France	758 400	2.5 (2.4 to 2.6)	15	2.5 (2.4 to 2.6)	18	0.0 (−0.2 to 0.2)	1.0 (0.9 to 1.1)
Germany	677 947	2.3 (2.2 to 2.4)	13	1.9 (1.8 to 2.0)	11	−0.4 (−0.5 to −0.2)^b^	0.8 (0.8 to 0.9)^b^
Hungary	90 335	3.5 (3.1 to 3.9)	24	3.4 (3.0 to 3.8)	25	−0.1 (−0.6 to 0.5)	1.0 (0.8 to 1.1)
Iceland	4907	1.2 (0.2 to 2.2)	2	1.2 (0.2 to 2.2)	2	0.0 (−1.4 to 1.4)	1.0 (0.3 to 3.1)
Israel	166 175	2.5 (2.3 to 2.7)	15	2.4 (2.2 to 2.6)	17	−0.1 (−0.4 to 0.3)	1.0 (0.8 to 1.1)
Japan	1 071 304	1.1 (1.0 to 1.2)	1	1.1 (1.0 to 1.2)	1	0.0 (−0.1 to 0.1)	1.0 (0.9 to 1.9)
Korea	470 171	1.8 (1.7 to 1.9)	8	1.8 (1.7 to 1.9)	8	0.0 (−0.2 to 0.2)	1.0 (0.9 to 1.1)
Latvia	19 781	3.4 (2.6 to 4.2)	23	3.6 (2.8 to 4.4)	27	0.2 (−1.1 to 1.3)	1.1 (0.8 to 1.5)
Netherlands	184 397	2.8 (2.6 to 3.0)	19	3.2 (2.9 to 3.5)	24	0.4 (0.1 to 0.8)	1.1 (1.0 to 1.3)
New Zealand	64 392	3.6 (3.1 to 4.1)	26	2.7 (2.3 to 3.1)	21	−0.9 (−1.5 to −0.3)^b^	0.8 (0.6 to 0.9)^b^
Norway	61 442	1.7 (1.4 to 2.0)	5	1.6 (1.3 to 1.9)	5	−0.1 (−0.5 to 0.4)	0.9 (0.7 to 1.4)
Poland	413 300	3.5 (3.3 to 3.7)	24	3.5 (3.3 to 3.7)	26	0.0 (−0.3 to 0.2)	1.0 (0.9 to 1.1)
Portugal	101 381	1.7 (1.4 to 2.0)	5	1.6 (1.4 to 1.8)	5	−0.1 (−0.5 to 0.3)	0.9 (0.8 to 1.2)
Slovak	60 410	3.6 (3.1 to 4.1)	26	3.6 (3.1 to 4.1)	27	0.0 (−0.7 to 0.7)	1.0 (0.8 to 1.2)
Slovenia	22 196	1.8 (1.2 to 2.4)	8	1.8 (1.2 to 2.4)	8	0.0 (−0.8 to 0.8)	1.0 (0.7 to 1.6)
Spain	486 575	2.1 (2.0 to 2.2)	11	2.1 (2.0 to 2.2)	13	0.0 (−0.2 to 0.2)	1.0 (0.9 to 1.1)
Sweden	115 641	1.6 (1.4 to 1.8)	4	1.3 (1.1 to 1.5)	3	−0.3 (−0.6 to 0.0)	0.8 (0.7 to 1.0)
Switzerland	80 290	3.1 (2.7 to 3.5)	22	2.6 (2.2 to 3.0)	20	−0.5 (−1.0 to 0.0)	0.8 (0.7 to 1.0)^b^
Taiwan	166 473	2.6 (2.4 to 2.8)	17	2.2 (2.0 to 2.4)	14	−0.4 (−0.1 to −0.0)^b^	0.9 (0.7 to 1.0)^b^
United Kingdom	804 667	3.0 (2.9 to 3.1)	21	2.5 (2.4 to 2.6)	18	−0.5 (−0.7 to −0.3)^b^	0.8 (0.8 to 0.9)^b^
US	3 999 386	4.1 (4.0 to 4.2)	28	3.1 (3.0 to 3.2)	23	−1.0 (−1.0 to −0.9)^b^	0.8 (0.7 to 0.8)^b^

Abbreviations: MT, minimum threshold; NMR, neonatal mortality rate.

^a^ NMR is the number of deaths per 1000 live births. The MT is defined as 22 weeks’ gestational age and a birth weight of 500 g.

^b^ The 95% CI for the difference between NMRs did not include 0.0, and the 95% CI for the ratio of NMRs did not include 1.0.

**Table 2.** NMRs, Country Ranks, Differences, and Ratios Between NMRs Without and With MTs for 26 Countries in 2020^a^

Country	Births, No.	Without MT		With MT		Difference between NMRs (95% CI)	Ratio of NMRs (95% CI)
		NMR (95% CI)	Rank	NMR (95% CI)	Rank		
Australia	294 369	2.4 (2.2 to 2.6)	17	2.4 (2.2 to 2.6)	20	0.0 (−0.2 to 0.3)	1.0 (0.9 to 1.1)
Austria	83 603	2.5 (2.2 to 2.8)	19	1.8 (1.5 to 2.1)	13	−0.7 (−1.1 to −0.3)^b^	0.7 (0.6 to 0.9)^b^
Belgium	114 350	2.2 (1.9 to 2.5)	15	2.0 (1.7 to 2.3)	16	−0.2 (−0.6 to 0.2)	0.9 (0.7 to 1.1)
Chile	193 488	4.3 (4.0 to 4.6)	26	4.0 (3.7 to 4.3)	26	−0.3 (−0.7 to 0.1)	0.9 (0.8 to 1.0)
Czechia	110 200	1.6 (1.4 to 1.8)	7	1.5 (1.3 to 1.7)	6	−0.1 (−0.4 to 0.3)	0.9 (0.7 to 1.2)
Denmark	61 240	2.0 (1.7 to 2.3)	13	1.6 (1.3 to 1.9)	10	−0.4 (−1.6 to 0.6)	0.8 (0.6 to 1.0)
Estonia	13 333	0.9 (0.4 to 1.4)	2	0.9 (0.4 to 1.4)	2	0.0 (−0.7 to 0.7)	1.0 (0.5 to 2.2)
Finland	46 463	1.3 (1.0 to 1.6)	3	1.4 (1.1 to 1.7)	5	0.1 (−0.5 to 0.5)	1.1 (0.8 to 1.5)
France	665 769	2.6 (2.5 to 2.7)	20	2.6 (2.5 to 2.7)	22	0.0 (−0.2 to 0.2)	1.0 (0.9 to 1.1)
Hungary	91 905	2.1 (1.8 to 2.4)	14	2.0 (1.7 to 2.3)	16	−0.1 (−0.5 to 0.3)	1.0 (0.8 to 1.2)
Iceland	4512	1.8 (0.6 to 3.0)	11	1.8 (0.6 to 3.0)	13	0.0 (−1.7 to 1.8)	1.0 (0.4 to 2.7)
Israel	177 260	1.6 (1.4 to 1.8)	7	1.5 (1.3 to 1.7)	6	−0.1 (−0.3 to 0.2)	0.9 (0.8 to 1.1)
Japan	840 835	0.8 (0.7 to 0.9)	1	0.8 (0.7 to 0.9)	1	0.0 (−0.1 to 0.0)	1.0 (0.9 to 1.1)
Korea	272 337	1.3 (1.2 to 1.4)	3	1.2 (1.1 to 1.3)	3	−0.1 (−0.3 to 0.1)	0.9 (0.8 to 1.1)
Latvia	17 552	2.4 (1.7 to 3.1)	17	2.3 (1.6 to 3.0)	19	−0.1 (−1.1 to 0.9)	1.0 (0.6 to 1.5)
Netherlands	168 681	2.9 (2.6 to 3.2)	22	3.4 (3.1 to 3.7)	25	0.5 (0.1 to 0.9)^b^	1.2 (1.0 to 1.3)^b^
Norway	53 461	1.3 (1.0 to 1.6)	3	1.3 (1.0 to 1.6)	4	0.0 (−0.4 to 0.4)	1.0 (0.7 to 1.4)
Poland	355 309	2.6 (2.4 to 2.8)	20	2.5 (2.3 to 2.7)	21	−0.1 (−0.3 to 0.2)	1.0 (0.9 to 1.1)
Portugal	84 426	1.7 (1.4 to 2.0)	9	1.6 (1.3 to 1.9)	10	−0.1 (−0.5 to 0.3)	0.9 (0.7 to 1.2)
Slovak	56 650	3.1 (2.6 to 3.6)	24	3.1 (2.6 to 3.6)	24	0.0 (−0.7 to 0.6)	1.0 (0.8 to 1.2)
Slovenia	18 514	1.4 (0.9 to 1.9)	6	1.5 (0.9 to 2.1)	6	0.1 (−0.7 to 0.8)	1.1 (0.6 to 1.8)
Spain	340 635	1.8 (1.7 to 1.9)	11	1.8 (1.7 to 1.9)	13	0.0 (−0.2 to 0.2)	1.0 (0.9 to 1.1)
Sweden	113 077	1.7 (1.5 to 1.9)	9	1.5 (1.3 to 1.7)	6	−0.2 (−0.5 to 0.1)	0.9 (0.7 to 1.1)
Switzerland	86 667	3.0 (2.6 to 3.4)	23	2.1 (1.8 to 2.4)	18	−0.9 (−1.4 to −0.4)^b^	0.7 (0.6 to 0.9)^b^
Taiwan	161 288	2.4 (2.2 to 2.6)	16	1.7 (1.5 to 1.9)	12	−0.7 (−1.0 to −0.3)^b^	0.7 (0.6 to 0.9)^b^
US	3 613 647	3.6 (3.5 to 3.7)	25	2.7 (2.6 to 2.8)	23	−0.9 (−0.9 to −0.8)^b^	0.8 (0.7 to 0.8)^b^

Abbreviations: MT, minimum threshold; NMR, neonatal mortality rate.

^a^ NMR is the number of deaths per 1000 live births. The MT is defined as 22 weeks’ gestational age and a birth weight of 500 g.

^b^ The 95% CI for the difference between NMRs did not include 0.0, and the 95% CI for the ratio of NMRs did not include 1.0.

Countries with significant differences showed improvements in their rankings. For example, in 2020, the ranking of NMR without and with an MT was 23rd and 18th for Switzerland, 19th and 13th for Austria, and 16th and 12th for Taiwan, respectively (Table 2). Furthermore, caution should be noted for countries with smaller numbers of deaths, because they had wider 95% CI ranges. In 2020, the NMRs ranked second through 10th did not significantly differ when considering the 95% CIs (Table 2).

## Discussion

The findings of this cross-sectional study indicate that some countries (6 in 2010 and 4 in 2020) exhibited significant changes in NMRs after the implementation of an MT. These countries may have a higher proportion of obstetricians adopting a definition-based approach,^2^ resulting in more periviable newborns, including many elective terminations of pregnancy, being registered as live births. Regarding countries showing the same NMRs with and without an MT, the OECD report^6^ indicates that these countries had set various registration criteria for live births in their official published neonatal and infant mortality rates.

One limitation of this study is that the exact number of neonatal deaths with an MT in each country was unavailable, and the estimated ones would be lower than the actual ones (eAppendix in Supplement 1). In conclusion, after the introduction of an MT to improve the comparability of NMRs across countries, the international variations in NMRs decreased. Furthermore, some countries exhibited greater outcomes than others.

## Notes

Supplement 1. **eAppendix.** Supplemental Methods
eReference

Supplement 2.
Data Sharing Statement
