# Supplementary material for: Neonatal Mortality Rates With and Without a Minimum Threshold
Source: JAMA Netw Open. 2024 Nov 22;7(11):e2447487. doi: 10.1001/jamanetworkopen.2024.47487 (PMC11584918; doi:10.1001/jamanetworkopen.2024.47487)
Supplement: Supplement 1. — eAppendix. Supplemental Methods eReference [file jamanetwopen-e2447487-s001.pdf]

Supplemental Online Content

Liang FW, Chang YS, Chou CS, Jen MY, Kawachi I, Lu TH. Neonatal mortality rates with and without a minimum threshold. *JAMA Netw Open*. 2024;7(11):e2447487. doi:10.1001/jamanetworkopen.2024.47487

eAppendix. Supplemental Methods

eReference

This supplemental material has been provided by the authors to give readers additional information about their work.

© 2024 Liang FW et al. *JAMA Network Open.*

eAppendix. Supplemental Methods

Additional data sources

OECD Health Statistics for 2010 and 2020 were obtained at https://www.oecd.org/en/data/datasets/oecd-health-statistics.html. The WHO Mortality Database can be found at https://www.who.int/data/data-collection-tools/who-mortality-database.

Selection of countries and study years

Not every OECD member country submitted data to OECD Statistics regularly and timely. For example, only 29, 25, and 21 countries submitted NMR data with a minimum threshold to OECD Statistics in 2010, 2020, and 2021, respectively, at the time of analysis for this study. 2010 is the earliest year in OECD Statistics for which both with and without a minimum threshold data were available, while 2020 was the latest year with the largest number of countries providing data with a minimum threshold.

Definition of neonatal mortality rate

Neonatal Mortality Rate (NMR) is defined as the number of neonatal deaths (deaths within the first 28 days of life) per 1,000 live births.

$$\mathrm{NMR} = \left(\frac{\text{Number of neonatal deaths}}{\text{Total number of live births}}\right) \times 1000$$

Formula for calculating 95% confidence intervals

The difference between NMRs with and without (w-w/o) a minimum threshold was calculated for each country using the following formula:

$$\text{Rate difference (RD)} = NMR_{\mathrm{w}} - NMR_{\mathrm{w/o}}$$

where NMR_{w} is the NMR with a minimum threshold and $NMR_{\mathrm{w/o}}$ is the NMR without a minimum threshold.

The 95% confidence interval (CI) for the rate difference was calculated using:

$$95\%\ \text{CI of RD} = \text{RD} \pm 1.96 \times \sqrt{\frac{NMR_{\mathrm{w}}}{N} + \frac{NMR_{\mathrm{w/o}}}{N}}$$

where N is the total number of live births.

The rate ratio of neonatal mortality rates (NMR) with a minimum threshold to that without a minimum threshold using the formula:

© 2024 Liang FW et al. *JAMA Network Open.*

$$\text{Rate ratio (RR)} = \left(\frac{NMR_{\mathrm{w}}}{NMR_{\mathrm{w/o}}}\right)$$

The corresponding 95% CI for the rate ratio was calculated using:

$$\text{95\% CI of RR} = \exp\left(\log(RR) \pm 1.96 \times \sqrt{\frac{1}{N_{with}} + \frac{1}{N_{without}}}\right)$$

where N_{with} and $N_{without}$ are the number of deaths in each NMR definition.

The exact number of neonatal deaths with a minimum threshold in each country was not available while searching the government vital statistics websites. Therefore, we estimated number of neonatal deaths with a minimum threshold using the number of births from the WHO Mortality Database and the NMR with MT obtained from OECD Statistics. The estimated number of neonatal deaths with threshold would be lower than the actual figure because the number of live births did not exclude births with gestational weeks lower than 22 weeks or birthweight lower than 500 grams. Consequently, the range of estimated 95% confidence intervals for rate ratios will be wider than the true values. This means that the significance tests of difference using 95% CIs in this study are more conservative than the actual values.

eReference:

Rosner B. Fundamentals of Biostatistics. 8th ed. Boston, MA: Cengage Learning; 2015.

© 2024 Liang FW et al. *JAMA Network Open.*
