# Supplementary material for: Neonatal Mortality Rates With and Without a Minimum Threshold
Source: JAMA Netw Open. 2024 Nov 22;7(11):e2447487. doi: 10.1001/jamanetworkopen.2024.47487 (PMC11584918; doi:10.1001/jamanetworkopen.2024.47487)
Supplement: Supplement 2. — Data Sharing Statement [file jamanetwopen-e2447487-s002.pdf]

Data Sharing Statement

Liang. Neonatal Mortality Rates With and Without a Minimum Threshold. *JAMA Netw Open*. Published November 22, 2024. doi:10.1001/jamanetworkopen.2024.47487

Data
Data available: Yes
Data types: Data (not involving human participants)
How to access data: Request for data please contact Tsung-Hsueh Lu robertlu@mail.ncku.edu.tw
When available: With publication

Supporting Documents
Document types: None

Additional Information
Who can access the data: Tsung-Hsueh Lu
Types of analyses: for any purpose
Mechanisms of data availability: without investigator support
Any additional restrictions: None
